# Extraction of Squalene From Tea Leaves (*Camellia sinensis*) and Its Variations With Leaf Maturity and Tea Cultivar

**DOI:** 10.3389/fnut.2022.755514

**Published:** 2022-02-10

**Authors:** Yue Yue Sheng, Jing Xiang, Kai Rong Wang, Ze Yu Li, Kai Li, Jian Liang Lu, Jian Hui Ye, Yue Rong Liang, Xin Qiang Zheng

**Affiliations:** ^1^Tea Research Institute, Zhejiang University, Hangzhou, China; ^2^Forest Technology Extension Center, Ningbo Agricultural and Rural Affairs Bureau, Ningbo, China

**Keywords:** *Camellia sinensis*, squalene, leaf maturity, GC-MS, saponification

## Abstract

Squalene is a precursor of steroids with diverse bioactivities. Tea was previously found to contain squalene, but its variation between tea cultivars remains unknown. In this study, tea leaf squalene sample preparation was optimized and the squalene variation among 30 tea cultivars was investigated. It shows that squalene in the unsaponified tea leaf extracts was well separated on gas chromatography profile. Saponification led to a partial loss of squalene in tea leaf extract and so it is not an essential step for preparing squalene samples from tea leaves. The tea leaf squalene content increased with the maturity of tea leaf and the old leaves grown in the previous year had the highest level of squalene among the tested samples. The squalene levels in the old leaves of the 30 tested cultivars differentiated greatly, ranging from 0.289 to 3.682 mg/g, in which cultivar “Pingyun” had the highest level of squalene. The old tea leaves and pruned littering, which are not used in tea production, are an alternative source for natural squalene extraction.

## Introduction

Squalene is a triterpene with molecular formula C_30_H_50_ [(6E,10E,14E,18E)-2,6,10,15,19,23-hexamethyl-2,6,10,14,18,22-tetracosahexaene], in which there are six isoprene units. Its density is 0.858 g/ml. Squalene is a precursor for biosynthesizing steroids in a wide variety of organisms and plays a crucial role in steroid synthesis in humans, especially dietary cholesterol (1, 2). Squalene is consisted of about 13% human skin surface lipids (3, 4), which protects the skin from lipid peroxidation caused by the adverse external environmental stresses such as ultraviolet radiation (5, 6). Squalene has attracted the great attention of researchers due to its diverse biological activities including antioxidant, anticancer, immune-boosting, antibacterial, and anti-inflammatory effects (2, 7–11), leading to a wide range of applications in the fields of food, pharmaceutical, and cosmetic industries.

Squalene was first identified in shark liver extract in 1903 (1, 12) and late was found in the other tissues of shark and vegetable oils such as olive oil (13). Although the liver of deep-sea sharks is an abundant natural source of squalene, excessive hunting endangered the shark species. The limited traditional natural resources (livers of the deep-sea sharks) call for alternative approaches to squalene isolation from plants and microorganisms. Partial inhibition of squalene epoxidase (SQE) in *Kluyveromyces lactis* cultivated on glucose and lactose media using specific inhibitor terbinafine (7.5 μg/ml) induced high accumulation of squalene in *K. lactis* cells, which is considered to be a promising way to produce natural squalene (14). Olive oil and amaranth oil are rich in squalene, with a mean content from 5.02 to 10.40 mg/g (15–19). However,the production volume of these vegetable oils is limited. The global production of olive oil was 308 million tons in 2019, which was only 2.5% of the total edible oil production of the world in the same year (20). The amaranth seed oil production is far less than olive oil. These suggest that olive and amaranth oils are not available in sufficient quantities for squalene extraction. Tea [*Camellia sinensis* (L.) O. Kuntze] is widely planted in many Asian and African countries and, tea leaves were found to contain squalene (21, 22) and it might be a source for extracting natural squalene. However, the concentration of squalene in tea leaves varied with the development of tea shoots (23). Chemical compositions of tea leaves varied with tea cultivars (23–26). However, the information on the squalene variation between tea cultivars has not been available yet. This study was set to investigate the difference in squalene content between leaves with various maturities and from various tea cultivars, which will be helpful to target the optimum source of tea materials for extracting natural squalene.

## Materials and Methods

### Plant Materials and Chemicals

All the plant materials used in this study were sampled from 12-year-old tea bushes (*C. sinensis*) grown in the Experimental Farm of Zhejiang University (Hangzhou, China) in September 2020. To investigate the effect of leaf development on squalene level, shoots with two leaves and an apex bud (the normal shoots for processing black tea and green tea), third leaf, fourth leaf, fifth leaf, sixth leaf, seventh leaf beneath the apex bud, and the old leaves are grown in the previous year were collected from plants of the cultivar ‘Jinxuan'. Each sample was collected from more than 100 bushes and 20 shoots each bush. The old leaves grown in the previous year from 30 tea cultivars were sampled from more than 100 bushes each cultivar for investigating squalene variation between tea cultivars. The deeply fermented tea (Liupao tea) used was supplied by Liubao Tea Corporation Ltd. (Wuzhou, China). All the tea leaves were dried to a moisture content of about 5% (W/W) in an oven at 80°C, ground and then sifted through a 24-mesh sifter. The ground samples were stored at −20°C till use. Squalene and amyrin references used were Sigma products (Sigma Aldrich Corporation, St. Louis, Missouri, USA). The n-hexane was purchased from Macklin Biochemical Corporation, Ltd. (Shanghai, China).

The moisture of the ground samples was determined by drying 1.00 g tea in an aluminum container at 105°C to a constant weight (the difference between the last two weightings was less than 1%). The moisture content of the tea samples was calculated based on the following formula:


$$\begin{array}{l} Moisture\;content\;\left(\%\right)=\frac{M1-Mn}{M1}\;*100 \end{array}$$

Here, M1 was the initial weight of the tea sample (g), Mn was the weight of the dried sample in the last weighting.

### Preparation of GC Reference Squalene

Squalene was diluted to 10 mg/mL using n-hexane which was used as stock solution. Concentration gradients of squalene from 0, 50, 100, 200, 400, 600, 800, 1,000, 1,500, and 2,000 μg/mL were prepared by diluting the stock solution with n-hexane.

### Extraction of Squalene From Tea Leaves

A total of 20 g of the ground tea sample was extracted in a 1,000-mL glass beaker with 200 mL n-hexane in a supersonic water bath at 40°C for 30 min. The residues were separated from the solution by filtration on a filter paper (No. 102 filter paper, Xinhua Paper Mill, Hangzhou, China) and then the residues were re-extracted two times as before. The filtrates were combined and evaporated at 40°C using a rotatory vacuum evaporator (Shenshun Biotechnology Corporation, Ltd., Shanghai, China) to a final volume of about 40 mL. The concentrated filtrate was transferred into a 50-mL volumetric flask and diluted to 50 mL using n-hexane.

### Saponification

The squalene extract from tea leaf or 1.0 mg/mL squalene n-hexane solution (50 mL) was saponified in a test tube containing 20 mL 12% KOH ethanol solution in the water bath at 60°C for 90 min according to the published method (22). When saponification was finished, the reacted solution was cooled to room temperature and mixed with double distilled water (20 mL) and n-hexane (20 mL) to isolate the unsaponifiable fraction. The mixed solution was allowed to stand for 30 min to obtain a clear separation of the two layers. The upper n-hexane layer was collected and the remained part was re-extracted once more as before. The collected n-hexane layers were transferred into a 100 mL volumetric flask, and diluted to a final volume of 100 mL with n-hexane.

### Gas Chromatography-Mass Spectrometry (GC-MS) Identification

Gas chromatography-MS qualitative and quantitative examinations of squalene in the tea leaf extracts were carried out using QP-2010 GC-MS with an AOC-20i autosampler (Shimadzu Corporation, Tokyo, Japan) according to the methods by Park et al. (22). The GC-MS conditions were as follows: DB-5 capillary column (0.25 mm × 0.25 μm × 30 m, Agilent Technologies, Foster City, California, USA), helium carrier gas with purity 99.999%, flow rate 1.0 mL/min, ion source temperature 230°C, inlet temperature 315°C, interface temperature 250°C, injection split ratio 20:1, injection volume 1 μL, oven temperature program: initial temperature 250°C for 1 min, then increasing to 300°C at 5°C/min, maintaining at 300°C for 18 min, scan rang 30–500 *m*/*z* with an even time 0.3 s and solvent delay time 2 min.

### Method Validation

The method validation was carried out based on the linearity of the standard curve, the limit of detection (LOD), the limit of quantification (LOQ), recovery, precision, and stability. For the recovery test, standard squalene solutions (0.5, 1.0, and 2.0 mg/g) were spiked to the tea samples before extraction and then extracted as described before. The recovery was calculated based on the difference in squalene content between the spiked sample and the non-spiked samples (background level in the tea leaves). The test precision of the method was validated using squalene gradient solutions (200, 400, and 800 μg/mL) for 3 repeated analyses. The stability of squalene solutions was validated by placing squalene gradient solutions (200, 400, and 800 μg/mL) at room temperature for 2, 4, 8, 12, and 24 h, and then they were determined by GC-MS as the above method. Precision and stability were expressed as % relative SD (% RSD).

### Statistical Analyses

The qualification analysis was based on the mass spectra of the compounds and the standard mass spectra provided by the NIST Mass Spectrometer Library. Quantificational analysis was done based on the retention time and peak area of the squalene reference. All the tests were repeated three times and the results were expressed as mean ± SD. Statistical analysis was performed using SPSS version 16.0 (SPSS, Chicago, IL, USA).

## Results

### Identification of Squalene in Tea Leaves

**Figure 1:**
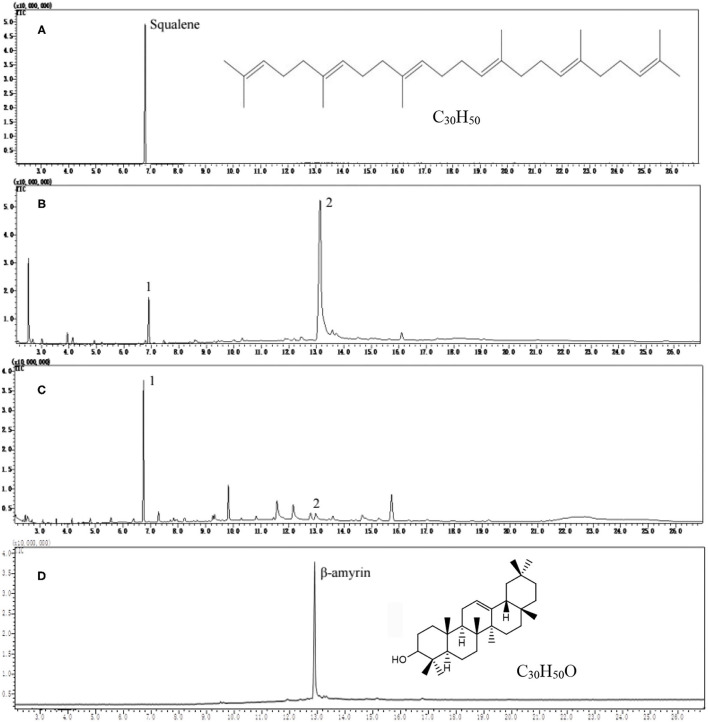
Gas chromatography (GC) profiles of squalene reference, tea leaf extracts, and β-amyrin reference. **(A)** Squalene reference, **(B)** tea extract after saponification, **(C)** tea extract before saponification, and **(D)** β-amyrin reference.

Squalene reference was well separated at retention time 6.85 min under GC conditions in this study (Figure 1A). However, great differences were observed in the GC profiles of tea extracts after saponification (AS) (Figure 1B) and before saponification (BS) (Figure 1C). In the saponified tea leaf extract, a strong peak 2 was observed at a retention time of 13.22 min except for peak 1 with the same retention time as squalene (Figure 1B). When the tea leaf extract without saponification was injected directly into GC, the peak 1 with a retention time as squalene was much stronger than that in the saponified tea leaf extract, but the peak 2 at retention time 13.22 min was very weak (Figure 1C). These suggest that saponification had a great impact on the GC profile of tea leaf extract.

**Figure 2:**
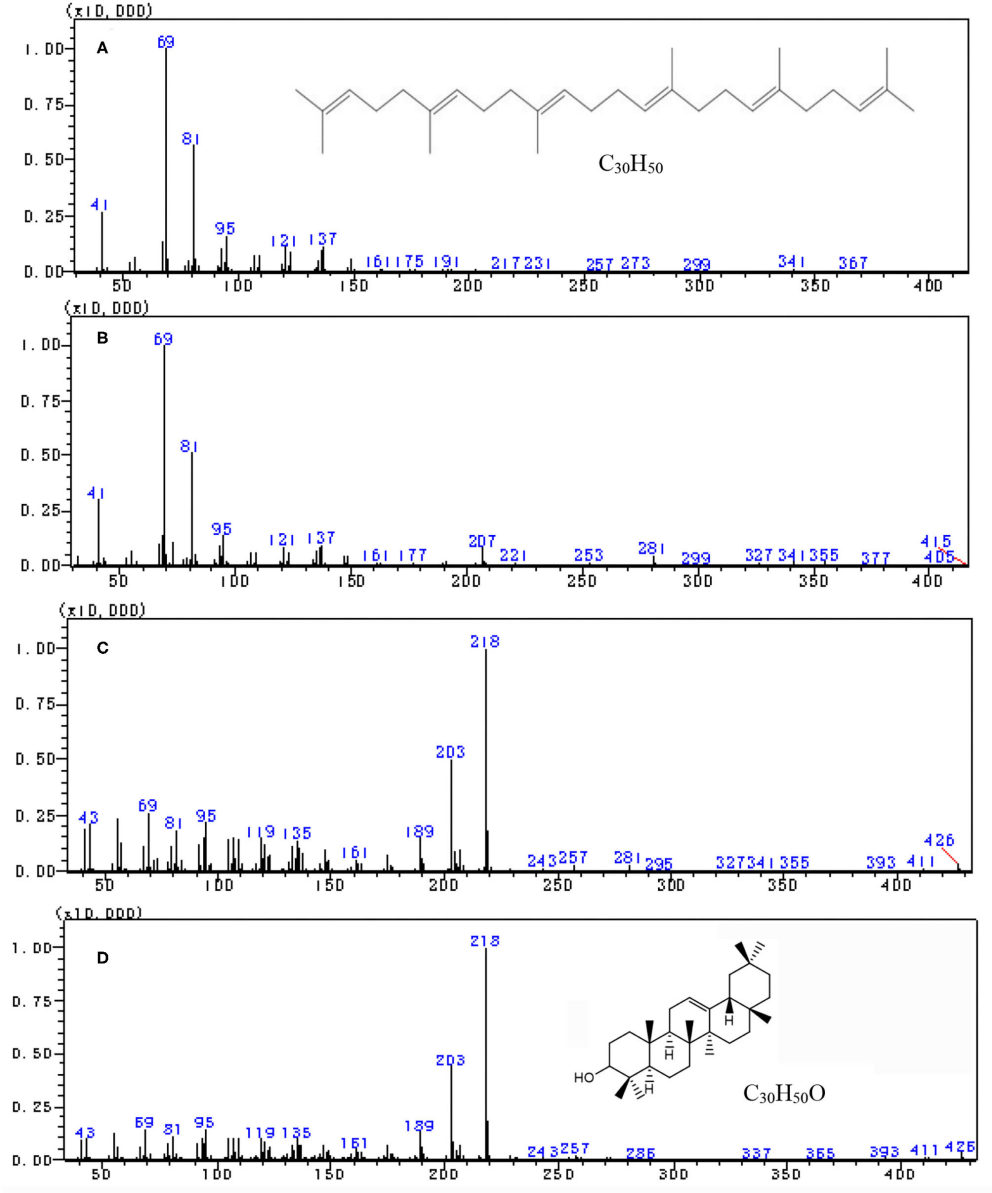
Full scan mass spectra by gas chromatography-mass spectrometry (GC–MS) analysis. **(A)** Squalene reference, **(B)** peak 1 in tea extract, **(C)** peak 2 in Figure 1B, and **(D)** β-amyrin reference.

The scan mass spectra showed that the mass spectra characteristics of peak 1 in Figure 1B were consistent with the authentic squalene, with the mass to charge ratios (*m*/*z*) of major fragments 69, 81, 41, 95, and total *m*/*z* of the parent ion 410 (Figures 2A,B), which were consistent with the results by Park et al. (22). Searching the NIST GC-MS Library (version NIST 14) revealed that the *m*/*z* of the peak 1 showed 97% similarity retrieval matching degree to squalene authentic compound (Figures 2A,B). Based on the retention time and the mass spectra characteristics, the peak 1 in Figure 1B was identified as squalene.

The peak 2 in Figure 1B was further analyzed by full scan mass spectra and it shows that the molecular mass of compound in peak 2 was 426, with major fragments at *m/z* 219, 203, 189, 69, and 95 (Figures 2C,D). Based on the searching results from the NIST GC-MS Library (version NIST 14), the peak 2 in Figure 1B showed 92% similarity retrieval matching degree to β-amyrin authentic compound (Figures 2C,D). The peak 2 in Figure 1B was identified as β-amyrin.

### Effect of Saponification on the Determination of Squalene in Tea Extracts

Figures 1B,C showed that saponification had a great impact on the GC profiles of tea extracts containing squalene. Though the GC profile of saponified tea extract was cleaner than that BS, the peak of squalene was much lower, and the peak of β-amyrin was much higher than that BS, respectively. Tests on old leaves harvested from cultivars ‘Jinxuan' and ‘Zhenong 113’ showed that 58 and 63% of squalene were lost during saponification based on the peak area of samples BS and AS, accompanied with an increase in β-amyrin by 29.75 and 42.57 times, respectively (Table 1). β-amyrin was increased AS through squalene was not detected in the Liupao tea, a deeply fermented tea produced in Wuzhou City, China. When pure squalene n-hexane solution (1 mg/ml) was saponified as tea extract, β-amyrin was not detected AS though the squalene was lost by 38% (Table 1). These indicate that the increase in β-amyrin was not induced by the loss of squalene. Figure 1C shows that the squalene in the un-saponified tea extract was well separated on the GC profile, suggesting that the saponification was not an essential step for GC determination of squalene in tea extracts. The tea extracts were directly injected into GC without saponification in the subsequent experiments.

**Table 1:** Effects of saponification on contents of squalene and β-amyrin (mean ± SD).

**Sample**		**Squalene**		**β-Amyrin**	
		**Peak area (Au)**	**AS/BS**	**Peak area (Au)**	**AS/BS**
Jinxuan^①^	BS	9121723.33 ± 2131.09	0.42 ± 0.01	1787188 ± 20089.92	29.51 ± 0.24
	AS	3860299.33 ± 62765.43		52737813.67 ± 226810.41	
Zhenong 113^②^	BS	36461692 ± 77292.72	0.38 ± 0.01	2644016 ± 50390.71	42.57 ± 1.40
	AS	13713970.33 ± 508631.58		112608236 ± 5876589.76	
Liupao tea^③^	BS	Nd	-	36445041.33 ± 629036.31	6.7 ± 0.08
	AS	Nd		244123254.33 ± 1214694.62	
Squalene^④^	BS	1301232471.33 ± 6118149	0.62 ± 0.01	Nd	-
	AS	812254139.33 ± 4968475.04		Nd	

① *, Old leaves of cultivar ‘Jinxuan’*;

② *, Old leaves of cultivar ‘Zhenong 113’*;

③ *, A deeply fermented tea produced in Wuzhou City, Guangxi, China*;

④ *, Squalene dissolved in n-hexane (1mg/mL); BS, Before saponification; AS, After saponification; Nd, non-detected*.

### Method Validation

**Figure 3:**
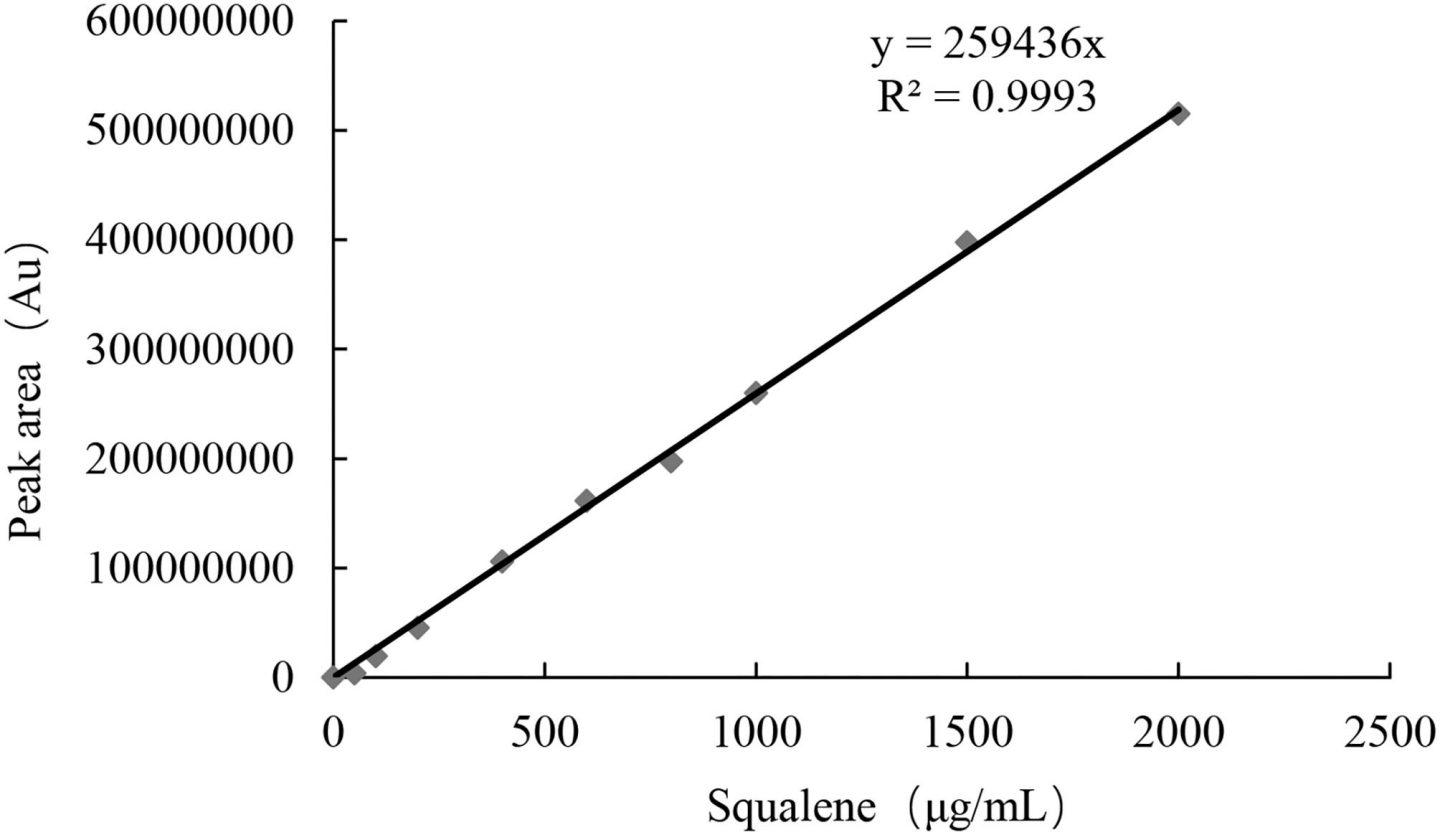
Linear regressive relationship between squalene concentration and peak area.

Test with a gradient concentration of squalene (0–2,000 mg/ml) revealed that the peak area showed a linear regressive relationship with the concentration of the squalene, with a linear correlation coefficient *R*^2^ = 0.9993 (Figure 3). The LOD and LOQ for squalene were 2.0 and 5.0 μg/ml, respectively (Table 2). The RSD between intraday injections decreased with the increase in squalene concentration, ranging from 5.68 to 1.46% RSD (Table 2). To investigate the stability of squalene during sample storage, squalene reference solutions at concentrations 200, 400, and 800 μg/ml were injected at 2, 4, 8, 12, and 24 h after preparation. It showed that the RSD of peak areas during 24 h storage of the reference solutions was less than 4.88%, suggesting that the squalene was basically stable during 24 h storage at room temperature (Table 3).

**Table 2:** Sensitivity and intra-day precision of the analytical method*.

**Sensitivity**		**Intra-day precision (RSD)**		
LOD (μg/mL)	LOQ (μg/mL)	200 (μg/ML)	400 (μg/ML)	800 (μg/ML)
2.00	5.00	5.68	2.27	1.46

* *: LOD, limit of detection; LOQ, limit of quantification, %RSD: relative standard deviation*.

**Table 3:** Changes in peak area (Au) during storage of squalene solutions.

**Concentration** **(μg/mL)**	**Storage time (h)**					**Mean ±SD**	RSD(%)*
	**2**	**4**	**8**	**12**	**24**		
200	58,312,545	62,072,252	64,032,482	61,403,130	56,707,498	60505581.33 ± 2643252.70	4.88
400	132,433,461	133,265,133	143,406,496	13,331,0423	140,000,486	136483199.83 ± 4407407.02	3.61
800	278,934,395	277,082,174	297,264,017	284,211,112	289,785,260	285455391.67 ± 7380701.80	2.89

When squalene was added to tea leaf containing 0.63 mg/g squalene (non-spiked sample) at concentrations 0.5, 1.0, and 2.0 mg/g, the recovery rates of the added squalene were 95.87, 111.75, and 93.86%, respectively, with RSD ranging from 1.90 to 5.03% (Table 4). These show that the present method for extracting squalene from tea leaves had a reasonable recovery rate.

**Table 4:** Results of recovery tests at three concentrations.

**Squalene added** **(mg/g)**	Non-spiked samples*(mg/g)	**Detected squalene (mg/g)**	**Recovery (%)**	RSD*(%)
0.50	0.63	1.11 ± 0.05	95.87 ± 4.78	5.03 ± 0.23
1.00	0.63	1.75 ± 0.01	111.75 ± 2.10	1.90 ± 0.05
2.00	0.63	2.51 ± 0.01	93.86 ± 2.11	2.24 ± 0.12

* *: Non-spiked samples: Background level of squalene; RSD, relative standard deviation*.

### Extraction Optimization

The extraction time and saponification were considered to be key factors influencing the extraction yield of squalene from plant materials (13, 22, 27). The accumulated extraction yield of squalene from tea leaves increased with the increase in extraction time, in which the content of squalene detected in the tea leaf increased quickly in the second extraction (Table 5). Taking the accumulated yield by four sequent extractions as 100%, about 72% of the squalene was extracted in the first extraction, the accumulated extraction rate of 1st and 2nd extractions was higher than 92%, and that of sequent three extractions was more than 97% (Table 5). In commercial extraction, taking into account the production cost and time, it is recommended to extract the tea leaves for two times based on the present results. However, in laboratory tests to assess the squalene concentration levels in tea leaves, consecutive three extractions are recommended.

**Table 5:** Accumulated content of squalene with extraction times (mg/g)*.

**Extraction time**	**1**	**2**	**3**	**4**
Before saponification	0.497 ± 0.012^f^ (71.82%)	0.637 ± 0.009^c^ (92.05%)	0.673 ± 0.000^b^ (97.25%)	0.692 ± 0.002^a^ (100%)
After saponification	0.437 ± 0.013^g^ (71.88%)	0.569 ± 0.001^e^ (93.59%)	0.595 ± 0.004^d^ (97.86%)	0.608 ± 0.002^d^ (100%)

* *: Data with different lowercase alphabetic letters were significantly different at p < 0.05. Data in the brackets were extract rates as the accumulated extraction rate of 1st to 4th extractions was set as 100%*.

### Changes in Squalene Content of Tea Leaves With Various Maturity

The content of squalene in the leaves on a tea shoot increased with the maturity of the leaf, ranging from 0.019 mg/g in the shoots with two leaves and an apex bud to 0.690 mg/g in the seventh leaf (Table 6). Furthermore, squalene in the old leaves grown in the previous year was even higher than that in the seventh leaf (Table 6). It indicates that the mature tea leaves grown in the present year or old tea leaves grown in the previous year are good materials for extracting squalene.

**Table 6:** Changes in squalene content with maturity of leaf*.

**Leaf position on shoots**	**Squalene (mg/g)**
Shoots with two leaves and a bud	0.019 ± 0.001^g^
3rd leaf	0.055 ± 0.001^f^
4th leaf	0.120 ± 0.011^e^
5th leaf	0.260 ± 0.004^d^
6th leaf	0.460 ± 0.015^c^
7th leaf	0.690 ± 0.021^b^
Old leaves	0.780 ± 0.021^a^

* *: Data with different lowercase alphabetic letters were significantly different at p < 0.05*.

### Changes in the Content of Squalene Between Various Tea Cultivars

Table 7 shows the changes in the content of squalene between 30 various tea cultivars. There were significant differences in the content of squalene between the tested tea cultivars, ranging from 0.289 to 3.682 mg/g. The old leaf of the cultivar ‘Pingyun’ contained the highest level of squalene (3.682 mg/g), followed by cultivars ‘Zhengnong 138’ (2.682 mg/g) and ‘Yingshuang’ (2.599 mg/g). Cultivar ‘Zhenong 21’ had the lowest level of squalene (0.289 mg/g) (Table 7). These suggest that cultivar is a very important factor influencing squalene concentration in tea leaves.

**Table 7:** Squalene content of various tea cultivars*.

**Cultivars**	**Squalene (mg/g)**
Pingyun	3.682 ± 0.047^a^
Zhengnong 138	2.682 ± 0.144^b^
Yingshuang	2.599 ± 0.089^b^
Meizhan	1.891 ± 0.029^c^
Longjingchangye	1.871 ± 0.069^c^
Hongyafoshou	1.829 ± 0.072^cd^
Zhenghedabaicha	1.821 ± 0.034^cd^
Zhenong 121	1.769 ± 0.016^d^
Qingxinqilan	1.750 ± 0.081^d^
Jiaming 1 Hao	1.651 ± 0.079^e^
Tengcha	1.603 ± 0.048^e^
Maoxie	1.495 ± 0.108^f^
Fenghuangshuixian	1.410 ± 0.041^fg^
Yunqi	1.401 ± 0.027^fgh^
Shuigucha	1.392 ± 0.048^gh^
Youxingshuixian	1.335 ± 0.086^ghi^
Yulan	1.302 ± 0.101^hi^
Zaohuangcha	1.259 ± 0.042^i^
Soubeizhong	1.124 ± 0.018^j^
Zhenong 113	0.932 ± 0.007^k^
Ribenpinzhong	0.895 ± 0.036^kl^
Ziyazhong	0.890 ± 0.034^kl^
Zhenong 139	0.802 ± 0.006^lm^
Xiangguliaobaihao	0.785 ± 0.027^m^
Fudingdabaicha	0.753 ± 0.007^mn^
Zhenong 12	0.680 ± 0.024^n^
Fujianshuixian	0.556 ± 0.004°
Luyafoshou	0.499 ± 0.018^°*p*^
Zisun	0.453 ± 0.020^p^
Zhenong 21	0.289 ± 0.005^q^

* *: Data with different lowercase alphabetic letters were significantly different at p < 0.05*.

## Discussion

Squalene is consisted of about 13% human sebum and plays an important role in quenching singlet oxygen in UV-associated skin damage (9). Squalene has multiple applications in the cosmetics, pharmaceutical, nutraceutical, and food industries owing to its unique physiological and functional properties (9, 28, 29). There was great commercial demand for squalene and the global squalene production volume was 2,500 tons annually, with a value of 93 million dollars in 2013, and is expected to reach 241.9 million dollars by 2022 (30). Squalene is traditionally produced by extracting livers of marine and freshwater fishes, and partially from vegetable oils (13, 31, 32), during which the squalene was extracted using saponification process followed with liquid–liquid extraction process (21, 33). Saponification refers to the reaction of alkali with an ester to produce alcohol and carboxylate, which was used to remove the saponifiable fraction so as to obtain a clean unsaponifiable fraction containing squalene (34). Strong bases such as sodium hydroxide and potassium hydroxide are generally used as base catalysts to react with the saponifiable fraction. The base catalyst has an impact on the yield of squalene. The use of potassium hydroxide as a base catalyst can produce a higher yield of squalene compared to sodium hydroxide (34). Theoretically, squalene will not be saponified. However, experiments showed that the content of squalene was significantly decreased AS (Figure 1 and Table 1). The decrease in squalene might be due to the structural damage of double bonds in squalene molecules during saponification since unsaturated bonds could be oxidized under alkaline conditions. Meanwhile, during alkali saponification and separation, a partial lye would be reacted with free fatty acids to generate soapstock, which absorbed partial squalene, leading to a decrease in the squalene content consequently (35). Figure 1 also shows that the squalene in the tea extract was well separated on the GC profile. It is concluded that saponification is not an essential process for preparing squalene samples from tea leaves with low-level oil through saponification is essential for separating the unsaponifiable fraction from the saponifiable fraction when squalene was isolated from fish livers and vegetable seeds containing a high level of oil.

**Figure 4:**
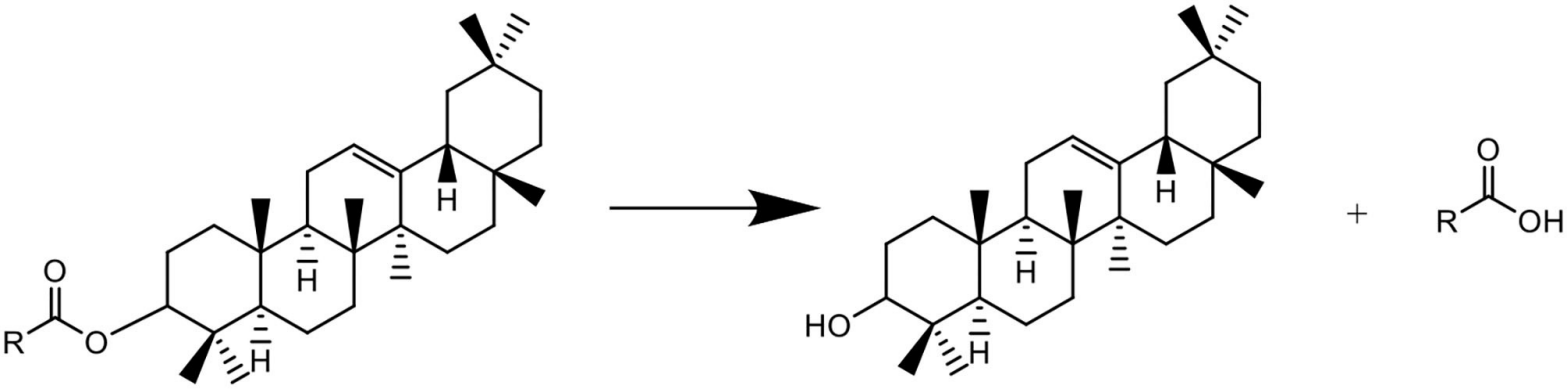
Putative formation of β-amyrin from β-amyrin ester during saponification.

In the biosynthesis pathways of triterpenes and sterols in the plant, β-amyrin is biosynthesized using squalene as substrate *via* intermediate oxidosqualene, which are catalyzed by SQE and oxidosqualene cyclase (36, 37). This process would not be happened in the present *in-vitro* experimental system, in which SQE and oxidosqualene cyclase are absent. This study shows that though the saponification of the extracts of freshly harvested tea leaves induced an increase in β-amyrin, accompanied with the decrease in squalene (Figures 1B,C and Table 1), the decrease in squalene was not the direct cause of the increase in β-amyrin because the content of β-amyrin was increased in the saponified extract of Liupao tea which contained no squalene, and furthermore, β-amyrin was not detected in the squalene solution AS (Table 1). It is presumed that in the matrix of the tea leaf, there might be acylated β-amyrins such as β-amyrin ester whose fatty acid motifs would be decomposed under the alkaline conditions during saponification, which led to the formation of β-amyrin (Figure 4). The possible reasons for the decrease of squalene AS might be due to: 1) squalene being partially oxidized under alkaline conditions and 2) squalene being partially transferred to the saponifiable fraction during the saponification and fraction partition.

This study reveals that squalene was increasingly accumulated in tea leaves with leaf maturity, in which the old leaves grown in the previous year contained the highest level of squalene (Table 6). These results are basically consistent with the findings by Park et al. (22). Squalene synthase (SQS) and SQE are key enzymes in squalene synthesis and metabolism, and they play key roles in the accumulation of squalene in microorganisms and plants. SQS-overexpression in *Chlamydomonas reinhardtii* (*C. reinhardtii*) increased the rate of conversion of C-14-labeled farnesylpyrophosphate into squalene, but did not lead to over-accumulation of squalene. Treatment of terbinafine, a specific inhibitor of SQE, caused the accumulation of squalene. SQE-knockdown reduced the expression level of SQE and increased the level of squalene in *C. reinhardtii* (38). Partial inhibition of SQE by terbinafine resulted in a high accumulation of squalene in yeasts *K. lactis* and *Saccharomyces cerevisiae* grown on glucose or lactose (14, 37–39). Ethylene promoted the post-ripening process of *Torreya grandis* (*T. grandis*) nuts, accompanying enhanced accumulation of squalene by inducing expression of genes in the mevalonate pathway such as 3-hydroxy-3-methylglutaryl-CoA synthase (HMGS) and 3-hydroxy-3-methylglutaryl-CoA reductase (HMGR). Exogenous ethylene treatment induced expression of squalene biosynthesis genes through several ethylene-responsive factors in post-ripening *T. grandis* nuts (40). Variations of SQS and SQE might be the reasons leading to the differences in squalene level between leaves with various maturity degrees and between tea cultivars. However, the molecular mechanism of squalene biosynthesis in tea plants is still unknown and further research is needed to better understand the accumulation of squalene in tea plants. Tender tea shoots with two leaves and a bud are usually used for processing black tea or green tea, but the old leaves grown in the previous year are not used in tea production. The significance of our findings is that the old tea leaves and the annually pruned littering which are not be used in tea production will be a good source of plant squalene. Furthermore, Table 7 shows that there was a significant difference in squalene content between tea cultivars, in which the highest one (cultivar ‘Pingyun’) was 12.7 times of the lowest one (cultivar ‘Zhenong 21’), suggesting that selection of cultivars plays a crucial role in using the old tea leaves as plant squalene sources.

Many vegetable oils were reported to contain squalene, such as rice bran oil (3.189 mg/g), hazelnut oil (0.279 mg/g), peanut oil (0.274 mg/g), and *Camellia* oil (0.110 mg/g) (41–45). *Amaranthus* is considered to be a rich source of plant squalene due to its high levels of squalene in seed (4.16 mg/g) and mature leaves (2.60 mg/g) (18, 45). This study shows that the squalene contents in old tea leaves from bushes of cultivars ‘Pingyun’, ‘Zhenong 138’, and ‘Yinshuang’ were 3.682 ± 0.047, 2.682 ± 0.144, and 2.599 ± 0.089 mg/g, respectively (Table 7), suggesting that old tea leaves and pruned littering from these tea cultivars will be an alternative source for plant squalene.

## Conclusion

In this study, methods for separating and determining squalene from tea leaves were optimized, and the variation of squalene content among leaves with various maturities and different tea cultivars were investigated. The results show that the extraction times and saponification treatment are important factors affecting the squalene content in the extract of tea leaves. The optimized method for extracting squalene from tea leaves is to extract the tea leaves using n-hexane as solvent (the ratio of leaf to solvent being 1/10, w/v) for three times, during which saponification of the leaf extracts is not an essential step. The squalene content in the tea extracts can be qualitatively and quantitatively detected by GC-MS. The foliar squalene content increased with the maturity of tea leaf and the old leaves grown in the previous year had the highest level of squalene. There was a great difference in squalene content between the tested tea cultivars, among which cultivars ‘Pingyun’ had the highest level of squalene (3.682 mg/g), followed by ‘Zhengnong 138’ (2.682 mg/g) and ‘Yingshuang’ (2.599 mg/g).

## Data Availability Statement

The original contributions presented in the study are included in the article/supplementary material, further inquiries can be directed to the corresponding authors.

## Author Contributions

KRW and XQZ contributed to funding acquisition. XQZ, JLL, and YRL contributed to project design and administration. YYS, JX, ZYL, KL, JLL, and JHY contributed to experimental work. YYS, JX, XQZ, and YRL contributed to the original draft preparation. YRL and XQZ contributed to review and editing. All authors approved the final version of the manuscript.

## Funding

This study was financially supported by the Ningbo Municipal Bureau of Science and Technology in the Major Project for Science and Technology Innovation of Modern Seed Industry 2025 (Project No. 2019B10022) and by the China Agriculture Research System of the Ministry of Finance of the People's Republic of China (MOF) and Ministry of Agriculture and Rural Affairs of the People's Republic of China (MARA).

## Conflict of Interest

The authors declare that the research was conducted in the absence of any commercial or financial relationships that could be construed as a potential conflict of interest. The reviewer LR declared a shared affiliation, though no other collaboration, with the authors to the handling editor.

## Publisher's Note

All claims expressed in this article are solely those of the authors and do not necessarily represent those of their affiliated organizations, or those of the publisher, the editors and the reviewers. Any product that may be evaluated in this article, or claim that may be made by its manufacturer, is not guaranteed or endorsed by the publisher.
